# WHO informal consultation on the guidelines for evaluation of the quality, safety, and efficacy of DNA vaccines, Geneva, Switzerland, December 2019

**DOI:** 10.1038/s41541-020-0197-2

**Published:** 2020-06-18

**Authors:** Rebecca Sheets, Hye-Na Kang, Heidi Meyer, Ivana Knezevic, WHO informal consultation on development of guidelines for assuring the quality, safety, and efficacy of DNA vaccine, Edward Abwao, Mohammed Alali, Patricia Aprea, Changjoon Bae, Carolina Damas Rocha Zarate Blades, Jean Boyer, Kate E. Broderick, Patrick Duffy, Aaron Farnsworth, Jayant Gangakhedkar, David Gutsch, Radwan A. Hafiz, Nick Jackson, Hye-Na Kang, David Kaslow, Amir S. Khan, Ivana Knezevic, Julie Ledgerwood, Margaret A. Liu, Joel Maslow, Heidi Meyer, Edwin Nkansah, Young Park, Ami Patel, Keith Peden, Trina Racine, Nicola Rose, Polly Roy, Rebecca Sheets, Manki Song, Wei Wei

**Affiliations:** 1 Independent expert, 20906 Silver Spring, MD USA; 2 World Health Organization, 1211 Geneva, Switzerland; 3 Paul-Ehrlich-Institut, 63225 Langen, Germany; 4 Pharmacy and Poisons Board, Nairobi, Kenya; 5 Alimentosy Tecnología Medica (ANMAT), Buenos Aires, Argentina; 6 Ministry of Food and Drug Safety, Cheongju, Republic of Korea; 7 Brazilian Health Regulatory Agency (ANVISA), Brasilia, Brazil; 8 Inovio Pharmaceuticals, San Diego, CA USA; 9 National Institutes of Health, Rockville, MD USA; 10 Health Canada, Ottawa, Canada; 11 Central Drugs Standard Control Organization (CDSCO), New Delhi, India; 12 Merck & Co, Inc, North Wales, PA USA; 13 Saudi Food and Drug Authority, Riyadh, Saudi Arabia; 14 Coalition for Epidemic Preparedness Innovations (CEPI), Oslo, Norway; 15 PATH - Vaccine Development Global Program, Seattle, WA USA; 16 National Institutes of Health, Bethesda, MD USA; 17 Karolinska Institute, Stockholm, Sweden; 18 GeneOne Life Science Inc, Blue Bell, PA USA; 19 Food and Drugs Authority, Accra, Ghana; 20 GeneOne Life Science Inc, Seoul, Republic of Korea; 21 The Wistar Institute, Philadelphia, PA USA; 22 US Food & Drug Administration, Silver Spring, MD USA; 23 Université Laval, Quebec City, QC Canada; 24 National Institute for Biological Standards and Control (NIBSC), South Mimms, UK; 25 London School of Hygiene & Tropical Medicine, London, UK; 26 Independent expert, Washington, DC USA; 27 International Vaccine Institute (IVI), Seoul, Republic of Korea; 28 National Medical Products Administration, Beijing, People’s Republic of China

**Keywords:** Infectious diseases, DNA vaccines

## Abstract

Consultations have been held to promote the revision of the WHO guidelines for assuring the quality and nonclinical safety evaluation of DNA vaccines adopted by the Expert Committee on Biological Standardization (ECBS) in 2005. The drivers for this revision are described, including the need for regulatory convergence highlighted by the WHO R&D Blueprint. These consultations have driven the revision to its current form, where a new guideline that includes quality, nonclinical, and clinical evaluation of plasmid DNA vaccines is being prepared for public consultation with a view to present to an upcoming ECBS. Major changes to the guidelines include streamlining the existing quality (part A) and nonclinical (part B) sections to reflect the two decades of experience, with manufacturing and control, nonclinical evaluation, and clinical testing of plasmid DNA vaccines, as a platform technology. The urgency for gaining regulatory convergence on this topic is that development of such a platform technology as DNA vaccines for routine use immunizations will prepare manufacturers and regulators across the globe in dealing with rapid development of medical countermeasures against emerging infectious diseases even in the face of an emergency setting. Two examples are described of Zika candidate vaccines that have rapidly advanced in development based on preexisting nonclinical and clinical data that precluded the need to repeat nonclinical toxicology. This report describes the progress stemming from the most recent consultation on the guidelines, including topics discussed and consensus reached.

## Introduction

Promoting regulatory convergence is recognized as a key enabler in the World Health Organization (WHO) R&D Blueprint. Regulatory preparedness for public health emergencies (PHEs) was on the agenda of the 17th International Conference of Drug Regulatory Authorities (ICDRA) meeting in 2016. A number of regulatory gaps were identified and ICDRA recommended WHO should ensure that regulatory support is a priority area of activity as the R&D Blueprint for emerging infectious diseases is implemented^1^. It was also requested WHO should continue developing measurement and written standards that serve as a basis for regulatory evaluation taking into consideration: (1) priority pathogens defined by the Blueprint, and (2) a more flexible and dynamic approach to developing and establishing standards for quality, safety, and efficacy of products for use in PHEs^2^.

In response to the request, WHO convened an informal consultation in February 2018 to initiate the work to revise the guidelines for assuring the quality and nonclinical safety evaluation of DNA vaccines (Annex 1, WHO Technical Report Series No. 941) adopted by the 2005 ECBS^3^. Based on the agreement in the informal consultation in February 2018, the first revised draft was prepared by a drafting group and posted on WHO Biologicals website for the first round of public consultation (https://www.who.int/biologicals/WHO_DNA_vaccine_HK_26_July_2019.pdf). The consultation in December 2019 aimed to discuss and obtain advice on the first draft document and main issues addressed from the public consultation.

About 35 experts participated in the consultation, including the regulators from 13 countries in six WHO regions.

Dr. Heidi Meyer (Paul-Ehrlich-Institut, Germany) was nominated as chairperson and Dr. Rebecca Sheets (WHO consultant, USA) as rapporteur for the consultation.

Dr Ivana Knezevic (WHO HQ, Switzerland) welcomed all the participants to Geneva and briefed the participants on the activities of WHO in the area of biological standardization. She explained that WHO is the directing and coordinating authority for health on behalf of the 194 member countries in the United Nations system. In order to fulfill WHO objectives, a core WHO function defined as setting norms and standards, and promoting and monitoring their implementation has been conducted for 70 years. This initiative includes assisting National Regulatory Authorities (NRAs) in the utilization of WHO Biological Reference Materials and application of the principles in WHO guidelines and recommendations, to ensure quality, safety and efficacy of vaccines, and other biologicals.

The world of immunization is a rapidly evolving field, and is constantly changing the picture of morbidity and mortality of infectious diseases. In that context, vaccines are playing a critical role in disease prevention and access to vaccines of assured quality is one of the goals of the WHO. At the same time, the use of new technologies for manufacturing, as well as new antigens, adjuvants, and routes of administration are imposing lots of challenges not only to regulators, but also to public health professionals. In addition, the response to PHEs, such as Ebola and Zika outbreaks triggered development of a number of vaccine candidates based on nucleic acid platforms. Clinical trials with these candidates for different diseases are either ongoing or planned in the near future (post meeting note: at the time of the submission of this paper for publication, more than ten vaccine candidates against the coronavirus disease (COVID-19), based on nucleic acids, are being developed. WHO is monitoring vaccines under development continuously and provides regular updates on it (https://www.who.int/blueprint/priority-diseases/key-action/novel-coronavirus/en/). This is increasing the importance of the revision and update of WHO guidelines for evaluation of DNA vaccines in line with the scientific advances.

Furthermore, WHO has other initiatives that are closely linked to the standardization of vaccines. In particular, strengthening of NRAs is one of the important elements in assuring the quality of vaccines worldwide. Prequalification of vaccines by WHO is an important mechanism through which vaccines become subject of supply by UNICEF. Safety of vaccines and the issues discussed at the WHO Global Advisory Committee on Vaccine Safety (GACVS) are also very important, as well as WHO activities related to immunization policy. In June 2019, the WHO GACVS set “six initial strategic priorities (which) are: systems and integration; equity and access; fragility and emergencies; values and ownership; research and innovation; and sustainability and accountability” (https://www.who.int/vaccine_safety/committee/reports/Jun_2019/en/). Of importance to the report herein are their priorities on research and innovation and on fragility and emergencies.

Dr Knezevic emphasized the importance of the present consultation that is the last step in terms of the face-to-face consultation on evaluating quality, safety, and efficacy of DNA vaccines, involving regulators, manufacturers, and other experts in this field. Representatives of several Collaborating Centers for biological standardization are also part of the consultation, as well as representatives of other institutions that are playing an important role in this field.

Dr Hye-Na Kang (WHO HQ, Switzerland) provided the background to the development of the guidelines and to the organization of this consultation. She reviewed the recommendations from the 17th meeting of ICDRA^1^ and the procedure of drafting guidelines by the WHO drafting group that consisted of Drs. Margaret A. Liu, Heidi Meyer, Edwin Nkansah, Keith Peden, Rebecca Sheets, and WHO secretariat Hye-Na Kang. In response to the request of the ICDRA, a drafting group prepared the first draft of document and released it for public consultation after a series of teleconferences (https://www.who.int/biologicals/WHO_DNA_vaccine_HK_26_July_2019.pdf). The objective of the informal consultation (December 2019) was to reach a consensus on the regulatory principles of the draft guidelines, discuss and identify any pending or critical issues such that an improved second draft can be prepared for the final round of public consultation, and adoption by the WHO ECBS in October 2020.

## Updates on DNA vaccine development

Several researchers in the field of plasmid DNA vaccines presented data on the current status of their development. There continues to be great interest by international groups on the promise of plasmid DNA vaccines as a platform technology for the rapid and facile development of vaccines to prevent outbreaks and pandemics. Dr. David Kaslow (PATH, USA) representing the WHO Product Development for Vaccines Advisory Committee (PDVAC) reviewed the advantages of DNA vaccines as a platform technology and how they fit into the objectives and goals of the PDVAC. He pointed out that the guideline revision may need to reflect the potential different purposes of a DNA vaccine—i.e., the use for routine immunization vs. the use for an outbreak setting. It will be important that DNA vaccines become available for the purposes of routine immunization if they are to be truly available in the case of outbreak response. He also raised some potential issues surrounding the need for a specific device for vaccine delivery, especially in the outbreak setting, when the supply may be limited and the cost-of-goods may not be favorable. In preparation for such situations, current device designs should be adapted for ease of use, and deployment in the setting of low- and middle-income countries (LMICs), as well as meet WHO programmatic suitability criteria for routine use. Dr. Kaslow also raised the issue of the need for more information on developmental toxicology for DNA vaccines to support their use in the setting of maternal immunization, including in breast-feeding women. These data are needed to pave the way for outbreak responses as pregnant women often belong to the group at highest risk for serious complications.

Dr. Nick Jackson (Coalition for Epidemic Preparedness and Innovation (CEPI), Norway) explained the role of the CEPI and their interest in plasmid DNA vaccines as a platform technology to achieve their mission. They target certain pathogens in their funding program, including Lassa virus, Middle East Respiratory Syndrome Coronavirus (MERS-CoV), Nipah virus, Rift Valley Fever virus, Chikungunya virus, and “Disease ‘X’” (a hypothetical template for an emerging disease of the future). A MERS-CoV plasmid DNA vaccine candidate has advanced into phase 1 clinical trials with promising results. They plan to initiate a phase 2 study in mid-2020. A Lassa virus candidate vaccine has also advanced into a phase 1 clinical trial.

Dr. Ami Patel (The Wistar Institute, USA) discussed many advantages of plasmid DNA vaccines and gave an update on a therapeutic Human Papilloma Virus vaccine candidate delivered in conjunction with an electroporation device. This vaccine candidate has also completed phase 2b pilot efficacy testing against cervical cancer, with very interesting results of 40–45% impact on endpoints, such as regression from cervical intraepithelial neoplasia (CIN) 2/3 to CIN 1, regression to normal, and clearance of evidence of infection^4^. A phase 3 study of this candidate opened in 2017. This candidate is also being studied in head and neck cancer^5^. Her team is also involved in development of Ebola, MERS-CoV, and Zika plasmid DNA vaccine candidates. In the case of Zika, her team was able to advance to the clinic with the candidate plasmid DNA vaccine rapidly (7 months) largely because of not needing to repeat nonclinical toxicology studies on their platform technology^6^. Instead, decisions were made on the basis of existing nonclinical and clinical data for the platform, which predict safe starting doses and dose regimens, as well as expected reactogenicity for the platform. Finally, she presented her work on expressing monoclonal antibodies (mAb) in the plasmid DNA system. Their anti-Zika candidate mAb has advanced to the clinic, as well.

An update was given by Dr. Julie Ledgerwood (NIH, USA) on a plasmid DNA vaccine candidate that had proceeded as far as an international phase 2b (pilot efficacy) study against Zika virus. While safety was demonstrated, the changing epidemiology in the face of a waning epidemic meant that too few cases of Zika were seen to evaluate efficacy; however, samples are still being analyzed and further cases may yet be identified. Importantly, because of their platform and earlier nonclinical and clinical work on a West Nile Virus plasmid DNA vaccine candidate, their Zika vaccine candidate was able to proceed to phase 1 in 3.25 months, in large measure because additional nonclinical toxicology studies were not required, abbreviating not only the nonclinical program, but the overall time-to-clinic. As with Dr. Patel’s team’s vaccine candidate, decisions were based on the existing database of nonclinical and clinical experience with the platform. Early entry into phase 1 permitted advancement to phase 2b even as the outbreak was ongoing. Unfortunately, this was still not rapid enough to capture sufficient efficacy data to support near-term licensure of the vaccine candidate^7^. She also presented data on studies performed earlier by her institute, in which plasmid DNA vaccine candidates for Ebola and Marburg were among the first plasmid DNA vaccines tested in Africa, demonstrating feasibility of testing these vaccines delivered by devices other than needle-and-syringe in LMIC settings^8^.

## Principles for evaluating of plasmid DNA vaccines

Plasmid DNA vaccines may be viewed as a platform technology in which only the antigen gene has been changed from one vaccine to another based on the same DNA plasmid backbone. For a given manufacturer, the manufacturing and controls may vary little between plasmid DNA vaccines they manufacture. Control measures are likely highly similar even between manufacturers. Therefore, the guidelines on plasmid DNA vaccines, though not specific to a particular vaccine, can be written so as to be generally applicable with part A, the quality section of the guidelines. In addition, although part B, nonclinical and part C, clinical sections will be consistent with the general guidelines on these topics, there are a few additional topic areas of relevance to plasmid DNA vaccines discussed in these guidelines^9–11^.

Many of the concerns that harken back to the beginnings of use of plasmid DNA vaccines, two decades ago, have been deleted from the guidelines as nonclinical and clinical evidence from various vaccine candidates over the intervening time have alleviated those concerns. The current generation of DNA vaccines made from bacteria are produced biologically and are considered to be a biological product. While the plasmid is generated by recombinant DNA technology, it should be clarified that a plasmid DNA vaccine itself is not an organism; thus, it is not a genetically modified organism per se, nor is it a gene-transfer or gene-therapy product, as it is not expected to persist and permanently “mark” the recipient. There is a wealth of evidence that DNA vaccines to date do not persist or even biodistribute throughout the body of the vaccine when delivered parenterally into muscle, subcutaneous tissue, or various dermal layers.

In addition, development of plasmid DNA vaccines for routine use may lead to rapid and ready implementation of new vaccines for emerging diseases even in the face of an emergency setting. Once the platform technology is proven safe and efficacious for one or more diseases, a novel vaccine candidate based on the same technology but replacing only the antigen encoded to match the emerging disease could permit rapid manufacturing, reduced (abbreviated) or waived requirements for nonclinical toxicology, and rapid entry into clinical testing. Rapid entry into the clinic has already been shown with two plasmid DNA vaccine candidates against Zika described above. Thus, proof-of-concept has precedent.

## Discussion on the draft guidelines

This session was dedicated to reviewing the draft guidelines in response to the public consultation. The discussion was led by Dr. Rebecca Sheets providing a summary of comments received from the first round of public consultation and main issues to be discussed at this meeting. Most of these comments were accepted and the changes made. Rejected comments were few, such as a suggestion to follow the ICH structure rather than the WHO standard. However, three topics remained for discussion by the consultants at the meeting. The first of these was surrounding several comments received on the discussion of potency assessment of multi-plasmid-containing vaccines. The second topic was about the appendix on heterologous prime-boost included in the current draft document. Several comments were made to delete the appendix as the information was included in the body of the guideline. The third topic was on a comment given to put more emphasis on the delivery device discussion.

The discussion on potency of multi-plasmid-containing vaccines led to a decision to clarify the discussion in the guideline for the rationale why this might need to be done at the individual bulk stage instead of in the final product. However, this discussion led to a more important and in-depth look at how potency is being assessed currently. Most vaccine candidates are moving forward into the clinical development without a bioassay, but simply measuring content (quantity—the basis for dosing) and percent supercoiled form of plasmid (a quality measure that should correlate with potency in vivo). This led to the discussion of revisions to the potency section itself. The appendix on heterologous prime-boost was agreed to be deleted. The discussion on the delivery device led to agreement that the information already in the general principles was mostly adequate, but that further emphasis on the labeling of what some jurisdictions might consider to be a combination product. It was also agreed that further discussion about the device in part C (clinical) would add further clarity to the guidelines.

During the consultation, several suggestions for amendment of the current draft document were made in order to reaffirm, clarify, and provide further guidance on specific issues. These are summarized in Table 1.

**Table 1:** The summary of major proposed changes in the revision of the draft guidelines for assuring the quality, safety, and efficacy of plasmid DNA vaccines.

Sections for revision	Proposed changes
Title of the document	Add “plasmid” to the title. Likewise, it was agreed to refer to DNA vaccines as plasmid DNA vaccines rather than DNA plasmid vaccines.
Terminology	Provide the definitions of adjuvant and candidate vaccine to align with the relevant WHO guidelines.
Scope	Plasmid DNA expressing prophylactic monoclonal antibodies were outside of scope, but that like some immunotherapeutics based on plasmid DNA, that the quality (part A) section may have applicability to this product class, though parts B (nonclinical) and C (clinical) are unlikely to apply.
Introduction	Add a few more of the advantages of plasmid DNA vaccines.
Part A, manufacturing and control	• Definition: the International Non-Proprietary Names conventions would apply to DNA vaccines. • General manufacturing guidelines: to clarify some language around potential for carryover or cross-contamination in multiuse facilities. • Source, history, and generation of the host cell and plasmid: to discuss the potential use of novel strains or species of bacteria, and to reflect more currently the means of assessing and ensuring genetic stability of the plasmid DNA construct. • Characterization of the bulk purified plasmid: clarification on characterizing the mode- or mechanism-of-action of the vaccine, including immunomodulatory elements. • Consistency of manufacturing: clarification to reflect that this part is not referring to clinical studies, but to timing of manufacturing assessments of consistency. • Manufacture and control of the final formulated vaccine: a subsection needed to be added to discuss measuring strength, dose, or content of the vaccine. • Potency: to change some language, as mentioned above, to reflect that potency might be measured by content and percentage supercoiling rather than use of a bioassay. Further clarity was given to measuring the expression by mRNA rather than protein production. • Safety, including sterility and endotoxin testing: to update to reference the monocyte activation test and to include the 3Rs concept.
Part B, nonclinical evaluation	• This section was considered to be generally satisfactory, though the need for more references that support the abbreviation of nonclinical programs would be useful to include for regulators in countries that have limited experience with this product type to date. Several references in this regard will be added. • Furthermore, some changes in part C made it apparent that further discussion was needed in part B on the subject of the existing database of biodistribution data showing limited distribution and rapid degradation outside the injection site, which assuage historical concerns about germ-line involvement or genetic transfer. Likewise, a need to acknowledge the current evidence gap in developmental toxicology for DNA vaccines was raised.
Part C, clinical evaluation	• It was agreed that this section required some additions and modifications. • Some language about the device/vaccine co-development should be included. The topics needed to be added included post-marketing surveillance issues, choice of control group (with or without use of device), and protocol/labeling language. • Further, an issue that seemed to have some potential to complicate or confuse safety evaluations was the timing of adverse events, following boost doses in heterologous prime-boost regimens and whether to attribute them to the prime or the boost.
Part D, guidelines for national regulatory authorities	• Language in part D, two official release and certification needs to be further examined since it is likely that licensing of a new plasmid DNA vaccine will be reflected in a product/disease-specific WHO guideline at the time of such. Therefore, it might not be accurate to say the vaccine lot would only be released if it fulfilled the part A, quality section of this broad DNA vaccine guideline. • Two appendices related to part D may need to be added to the guidelines. – Model summary protocol for the manufacturing and control of DNA vaccines. – Model national regulatory authority lot release certificate for DNA vaccines.

## Conclusion and way forward

- The development of guidelines was welcomed and applauded by stakeholders.
- Participants agreed that the guidelines incorporate sound scientific evaluation principles consistent with international initiatives and would promote regulatory convergence.
- It might be necessary for WHO to assist NRAs in implementing the principles of the guidelines into their regulatory practices.
- Due to rapid technological advances in DNA vaccine development, it was proposed that there should be more frequent updating of these guidelines.
- WHO support to develop guidelines on regulatory evaluation of vaccines for PHE uses, as well as of combination products was requested.
- It was agreed that the guidelines be revised based on the comments received. Following revision and another round of public consultation, the document will be discussed at the meeting of WHO ECBS in October 2020 for adoption.
- A need for facilitating implementation of updated WHO guidelines for evaluation of DNA vaccines was identified as an activity that would help NRAs in WHO members states to establish and/or update national guidance for DNA vaccines.
- The consultation also recognized that development of RNA-based vaccines requires WHO action. It was proposed to consider preparation of a State of the Art Paper on the evaluation of RNA-based vaccines (Table 1). It was clear that the scientific evidence for these vaccines is limited and more data will most likely become available in coming years (Table 1). Nevertheless, the importance of WHO leadership in this area was identified as one of the follow-up actions beyond the revision of WHO guidelines for evaluation of DNA vaccines (Table 1).

### Reporting summary

Further information on research design is available in the Nature Research Reporting Summary linked to this article.

## Supplementary information

Reporting Summary
